# Composition of Stallion Seminal Plasma and Its Impact on Oxidative Stress Markers and Spermatozoa Quality

**DOI:** 10.3390/life11111238

**Published:** 2021-11-16

**Authors:** Filip Tirpák, Marko Halo, Katarína Tokárová, Lukasz J. Binkowski, Jaromír Vašíček, Andrea Svoradová, Martyna Błaszczyk-Altman, Anton Kováčik, Eva Tvrdá, Peter Chrenek, Norbert Lukáč, Peter Massányi

**Affiliations:** 1 AgroBioTech Research Centre, Slovak University of Agriculture in Nitra, Tr. A. Hlinku 2, 949 76 Nitra, Slovakia; marko.halo1@uniag.sk; 2 Institute of Applied Biology, Slovak University of Agriculture in Nitra, Tr. A. Hlinku 2, 949 76 Nitra, Slovakia; katarina.tokarova@uniag.sk (K.T.); anton.kovacik@uniag.sk (A.K.); eva.tvrda@uniag.sk (E.T.); norbert.lukac@uniag.sk (N.L.); peter.massanyi@uniag.sk (P.M.); 3 Institute of Biology, Pedagogical University of Krakow, Podchorazych 2, 30-084 Krakow, Poland; lukasz.binkowski@up.krakow.pl (L.J.B.); martyna.blaszczyk-altman@up.krakow.pl (M.B.-A.); 4 Institute of Biotechnology, Slovak University of Agriculture in Nitra, Tr. A. Hlinku 2, 949 76 Nitra, Slovakia; jaromir.vasicek@uniag.sk (J.V.); peter.chrenek@uniag.sk (P.C.); 5 NPPC—Research Institute for Animal Production Nitra, Institute of Farm Animal Genetics and Reproduction, Hlohovecka 2, 951 41 Luzianky, Slovakia; svoradovaandrea1@gmail.com; 6 Department of Morphology, Physiology and Animal Genetics, Mendel University in Brno, Zemědělská 1665/1, 613 00 Brno, Czech Republic

**Keywords:** horse, prooxidant activity, antioxidant activity, micro and macroelements, spermatozoa, DNA

## Abstract

The composition of seminal plasma of individual sires varies and so does the fertilizing ability. Micro and macro elements along with seminal enzymes, hormones, proteins, and lipids contained in seminal plasma are essential for the proper physiological function of spermatozoa. However, elevated levels against the normal physiological values, especially in the case of trace metals, result in the production of reactive oxygen species. The deficiency of antioxidants in the seminal plasma that could scavenge free radicals causes an impairment of spermatozoa quality. Ejaculates were obtained from 19 stallions. The fresh semen was analyzed to evaluate qualitative parameters of spermatozoa in terms of the motility, viability, and integrity of DNA. Separated seminal plasma underwent the assessment of the chemical and biochemical composition and RedOx markers. Based on the obtained concentrations of individual chemical elements, the correlation analysis suggested a negative impact of Cu in seminal plasma on the SOD, GPx, and LPO. Contrary, positive correlation was detected between FRAP and motility features. While Cu negatively correlated with sperm motion parameters, the adverse effect on viability was suggested for Cd. Our data suggest that seminal plasma has a potential due to its availability to become the potential biomarker of the reproductive health of farm animals.

## 1. Introduction

Seminal plasma (SP), the product of testes, epididymides, and accessory sex glands, is a fluid released during ejaculation and representing up to 98% of the voluminous stallion ejaculate. In horses, the process of ejaculation is composed of six to nine fractions of semen, which vary in chemical composition [1]. SP plays an important role in reproduction not only as a transportation medium, but also as a source of energy [2], antioxidants [3], enzymes [4,5], and minerals [6].

Micro and macro elements in SP directly contribute to the sperm quality. Calcium and phosphorus maintain the functional integrity of the spermatozoa [7]. Moreover, the positive effect of calcium on the sperm motility was reported by Halo et al. [6] and Marzec-Wroblewska et al. [8]. Magnesium, more concentrated than in the blood plasma, ensures the activity of seminal enzymes. Contrary, a low level of magnesium is associated with premature ejaculation [9]. Some trace elements such as zinc, selenium, iron, etc., avoid the general bias about the necessarily negative effect of heavy metals. Even metals in specific trace amounts contribute to essential physiological and biochemical processes [10]. Kerns et al. [11] present zinc ion (Zn^2+^) as a crucial factor in reproduction, being involved in capacitation. Zinc along with copper, iron, manganese, and nickel are the main metal cofactors forming three major families of superoxide dismutase (SOD), which belongs among the most abundant antioxidant enzymes in mammalian seminal plasma [3]. SOD together with catalase (CAT), glutathione peroxidase (GPx), and glutathione reductase (GSR) are engaged in the scavenging of reactive oxygen species (ROS) [12]. Oxidative stress (OS), a well-recognized aspect impairing male fertility, is the consequence of a disbalance between ROS and the antioxidant capacity. Excessive exposure to ROS may result in the degradation of biomolecules, alteration of androgen production, and an overall aggravation of fertility [13,14,15]. As every single aerobic cell, spermatozoa are challenged by the so-called “Oxygen Paradox”. Even though oxygen is a serious threat for the gametes, at the same time, it is inevitable for the physiological cell functions. Moreover, a low level of ROS is crucially involved in the spermatozoa capacitation, hyperactivation, acrosome reaction, and sperm–egg fusion [16]. The importance of the liquid part of the semen is proved not just in the male reproductive tract but also in the female reproductive system [17]. Seminal plasma interacts with the female genital tract epitheliums and their secrets. Seminal proteins initiate an inflammatory reaction that leads to the cleaning of the intrauterine cavity from foreign cells and pathogens, thus preparing the uterus for embryo implantation. It is also presumed that SP proteins contribute to higher embryo survival and fertility [18]. On the other hand, SP may have a deteriorative effect on processed spermatozoa. Akcay et al. [19], Alghamdi et al. [20], and Brinsko et al. [21] reported that the storage of stallion spermatozoa with SP in cooled or frozen form decreases spermatozoa quality in terms of viability, membrane integrity, and motility. Therefore, it is necessary to distinguish between the effects of SP on spermatozoa during the natural mating or in the process of artificial insemination.

Semen reflects besides genetic predispositions, diet, paternal effects, and sexual maturity also the state of the environment [22]. Toxic wastes in the form of endocrine disruptors [23] or heavy metals [24], as a result of the excessive industrial and anthropogenic activity accumulate in the biosphere [25,26,27], which eventually leads to the contamination of the fauna and flora [28,29,30,31,32,33,34]. Thus, chronic intoxication of the domestic animals, displayed in the impaired fertility, may occur even in a seemingly safe habitat. Due to the relatively high sensitivity of the reproductive tract, semen may be considered the indicator of overall and reproductive health [35]. As such, the spermatozoa of sires should be examined before every mating season, in such a way that the composition of SP should be monitored for the presence of enhanced or decreased levels of its components with special emphasis on the presence of toxic elements and other pollutants.

The present study aimed to determine the concentration of micro and macro elements (Ca, Cd, Cu, Fe, Hg, K, Na, Mg, Pb, and Zn) and to define the protein and lipid profile of the stallion SP. The current state of OS markers was monitored to predict the effect of the seminal fluid constituents on the OS and qualitative spermatozoa parameters via correlation analyses. The current study points to the importance of the seminal plasma as a specimen for the evaluation of the reproductive health of breeding stallions.

## 2. Materials and Methods

### 2.1. Semen Collection and Processing

Semen samples were obtained from clinically healthy breeding stallions (*n* = 19) in western Slovakia (District of Nitra) in the age of 8–19 years composed of the following breeds: Lipizzaner, Hucul, Oldenburger, and Arab thoroughbred. Stallions subjected to the study are fed and housed under the same conditions and routinely undergo semen collection during the breeding season with a frequency of collections of three times per week. The animals were carefully handled in accordance with the ethical guidelines of the Slovak Animal Protection Regulation RD 377/12, conforming to European Union Regulation 2010/63. Semen was collected within two weeks in April using a lubricated pre-warmed artificial vagina (Colorado model, Minitüb, Landshut, Germany) following stimulation of stallion by a mare situated close to the breeding phantom. Ejaculates were immediately split into two aliquots. The first one was used for the analyses of the sperm motility, viability, mitochondrial activity, and DNA fragmentation. The seminal plasma of the other aliquot was separated by the centrifugation which took place on the site of the collection and was carried out at 3000× *g* for 10 min. Accordingly, centrifugation was conducted one more time with the supernatant of the first centrifugation for the thorough separation of cell fraction from seminal plasma [36].

### 2.2. Motility Analysis

Spermatozoa were analyzed at time 0 and after 1 h storage at 6 °C. Each sample was placed into a Makler Counting Chamber^®^ (depth 10 μm, Sefi–Medical Instruments, Haifa, Izrael). Using the species-specific set-up, the basic and the most important parameters were selected—total motile spermatozoa (MOT; %), progressively motile spermatozoa (PRO; %), velocity curved line (VCL; µm/s), amplitude of lateral head displacement (ALH; µm), and beat cross frequency (BCF; Hz)—and were evaluated using the computer-assisted semen analyzer (CASA) Sperm Vision^®^ program (Minitube, Tiefenbach, Germany) equipped with a microscope (Olympus BX 51, Olympus Corporation, Tokyo, Japan) [37]. Within each of the CASA assessments, several different fields of view of Makler Counting Chamber were evaluated as described by Slanina et al. [38] and Tirpak et al. [39]. To distinguish the time of the analysis, every parameter is labeled with an underscore (_) and the time of the measurement (0—initial analysis; 1—analysis after an hour incubation at 6 °C) (e.g., MOT_0, MOT_1, etc.).

### 2.3. Detection of Apoptosis

**Figure 1:**
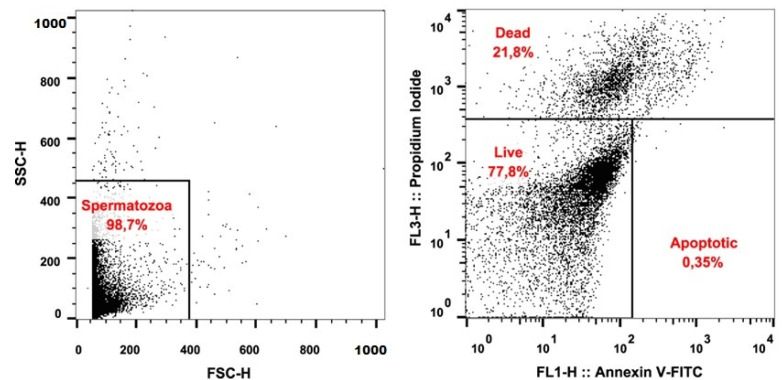
Illustrative dot plots used for the flow-cytometric evaluation of the stallion spermatozoa apoptosis: Live (AnV^−^/PI^−^), apoptotic (AnV^+^/PI^−^), and dead (AnV^−^/PI^+^ and AnV^+^/PI^+^) spermatozoa.

The flow-cytometric method was employed to identify the apoptosis of spermatozoa. For this purpose, fluorescent probes Annexin V-FITC (Annexin V Apoptosis Detection Kit, Canvax, Cordoba, Spain) and propidium iodide (PI; Molecular Probes, Eugene, OH, USA) were used. The assay was performed following the method described by Kuzelova et al. [40] with slight modifications. Briefly, the semen samples were washed and centrifugated in Dulbecco’s phosphate-buffered saline + Ca; Mg (DPBS + Ca; Mg, Gibco, Waltham, MA, USA) at 600× *g* for 7 min. The supernatant was discarded, and aliquots of 10^6^ cells were used for staining. Annexin V staining was done according to the manufacturer’s instructions. Immediately prior to analysis, 4 μL of the PI were added to the cell suspension. The cells were evaluated using FACS Calibur^TM^ (BD Biosciences, Franklin Lakes, NJ, USA) and Cell Quest Pro^TM^ (BD Biosciences, USA) software. A minimum of 10,000 cells was analyzed for each sample. Cells found in the lower left quadrant (AnV^−^/PI^−^) were identified as live. Cells located within the lower right quadrant (AnV^+^/PI^−^) were determined as apoptotic. Both upper quadrants (AnV^−^/PI^+^ and AnV^+^/PI^+^) included dead sperm cells [41]. The evaluation of spermatozoa viability is illustrated in Figure 1.

### 2.4. Assessment of Sperm DNA Integrity

Sperm DNA fragmentation was assessed using commercially available kit Dyn-halosperm^®^ (Halotech DNA, Madrid, Spain). Tubes containing aliquots of low-melting-point agarose were placed in a water bath at 90–100 °C for 5 min to fuse the agarose and subsequently transferred to an incubator at 37 °C. After 5 min of incubation, 20 μL of the sample was mixed with the fused agarose. Twenty microliters of the semen-agarose mix were pipetted onto slides pre-coated with agarose and covered with a 20 × 20 mm coverslip. Then, the slides were placed on a cold plate at 4 °C for 5 min to allow the agarose to turn into a microgel with the spermatozoa embedded within. The coverslips were gently removed, and the slides were immediately immersed horizontally into an acid lysis solution for 5 min. After washing in distilled water for 5 min, the slides were dehydrated in 70% and 100% ethanol for 2 min each and finally air-dried. All slides were stained using SYBR Green (2 μg/mL) (Sigma-Aldrich, St. Louis, MO, USA) in VECTASHIELD (Vector Laboratories, Burlingame, CA, USA) and a minimum of 300 spermatozoa per sample was scored using an Epifluorescence Leica DMI6000 microscope using a dry ×40 fluorite magnification objective (Leica Microsystems, Wetzlar, Germany). The incidence of DNA fragmented spermatozoa was expressed in percentages (%) [42].

### 2.5. Mitochondrial Activity

The viability of the spermatozoa was assessed using the mitochondrial toxicity test (MTT). This colorimetric assay measures the conversion of 3-(4,5-dimetylthiazol-2-yl)-2,5-diphenyltetrazolium bromide (Sigma-Aldrich, St. Louis, MO, USA) to purple formazan particles by mitochondrial succinate dehydrogenase of intact mitochondria of living cells. Optimal density was determined spectrophotometrically at a wavelength of 570 against 620 nm as a reference by a microplate ELISA reader (Multiskan FC, ThermoFisher Scientific, Vantaa, Finland). The data were expressed as absorbance (Abs) [43,44].

### 2.6. Detection of Micro and Macro-Elements in Seminal Plasma

All samples (0.5 mL each) were mixed with 1 mL of nitric acid (65%, Baker Analyzed, JT Baker, Phillipsburg, NJ, USA) and mineralized in an open mineralization system (Velp Scientifica DK20, Usmate Velate, Italy) at 140 °C. Then, the obtained solutions were diluted with ultrapure water (18.2 MΩ-cm at 25 °C, Direct-Q 3, Merck-Millipore, Germany) up to 10 mL [45]. The concentrations of chemical elements (Ca, Cd, Cu, Fe, K, Na, Mg, Pb, and Zn) in seminal plasma were analyzed with a flame atomic absorption spectrometer (AAnalyst 200, PerkinElmer, Waltham, MA, USA). Limits of quantification (LoQ) established for semen samples were Ca (0.514 mg/L), Cd (0.010 mg/L), Cu (0.035 mg/L), Fe (0.415 mg/L), K (0.445 mg/L), Na (0.045 mg/L), Mg (0.017 mg/L), Pb (0.107 mg/L), and Zn (0.024 mg/L).

Total mercury concentration (Hg) was measured using a cold vapor atomic absorption spectrometer MA−2 (Nippon Instruments Corporation, Bukit Batok, Singapore) in 100 µL of each sample (with two repetitions; the mean value was used for further analyses if the RSD between replicates was lower than 10%, and if not, the sample was reanalyzed) [34]. The limit of quantification for Hg was 0.02 μg/L.

### 2.7. Ferric Reducing Ability of Plasma (FRAP)

The FRAP analysis was performed according to Benzie and Strain (1999) and modified by Tvrda et al. [46]. It is a straightforward test to determine the total antioxidant power, based on the antioxidant driven colorimetric change caused by the reduction of a ferric-tripyridyl triazine complex to ferrous colored form. Calibration was realized using solutions of FeSO_4_·7 H_2_O. A working reagent was pipetted into a 96-well micro plate, and a reagent blank reading was conducted at 593 nm using a Promega GloMax Multi+ Microplate Reader (Madison, WI, USA). After that, standards and samples were added. Second absorbance was read after 4 min, and the results of FRAP was calculated using the standard curve and expressed in FeSO_4_·7 H_2_O.

### 2.8. Superoxide Dismutase (SOD)

SOD was analyzed by the RANDOX assay kit RANSOD (Randox Laboratories, Crumlin, UK) according to the manufacturer’s instructions using Randox RX Monza (Randox Laboratories, Crumlin, UK). The method for measuring SOD employs xanthine and xanthine oxidase (XOD) to generate superoxide radicals, which react with 2-(4-iodophentyl)-3-(4-nitrophenol)-5-phenyltetrazolium chloride to form a red formazan dye. SOD activity was measured by the inhibition degree of this reaction at 505 nm and expressed as U/mg of total protein.

### 2.9. Glutathione Peroxidase (GPx)

GPx activity was analyzed by the RANDOX assay kit RANSEL (Randox Laboratories, Crumlin, UK) according to the manufacturer’s instructions using Randox RX Monza (Randox Laboratories, Crumlin, UK). Enzyme activity was recalculated and expressed as U/mg of total protein. The method for measuring GPx is based on the catalyzation of glutathione by cumene hydroperoxide. In the presence of glutathione reductase and nicotinamide adenine dinucleotide phosphate, the oxidized glutathione is immediately converted to the reduced form with the concomitant oxidation of NADPH to NADP^+^. The decrease in absorbance at 340 nm is measured [47].

### 2.10. Total Oxidant Status (TOS)

The principle of TOS analysis is based on the oxidation of ferrous ions-o-dianisidine complexes by the oxidants present in the sample to ferric ions. The process of oxidation reaction was supported by the glycerol molecules present in the reaction solution. Then, the ferric ions formed a colored complex with xylenol orange in the acidic environment of the reaction solution. The color intensity, which can be measured spectrophotometrically, corresponds to the total amount of oxidant molecules present in the sample. The assay is calibrated using hydrogen peroxide, and the results are expressed as μmol H_2_O_2/_L (Erel, 2005). Briefly, we prepared reaction solutions 1 and 2 (TOS R1 and TOS R2). The TOS R1 consisted of 150 μmol xylenol orange disodium salt (Sigma-Aldrich, St. Louis, MO, USA), 140 mmol sodium chloride (Sigma-Aldrich, St. Louis, MO, USA), and 1.35 mol glycerol (Centralchem, Bratislava, Slovakia) in 25 mmol H_2_SO_4_ (Sigma-Aldrich, St. Louis, MO, USA). The TOS R2 was composed of 5 mmol ferrous ammonium sulfate hexahydrate (Centralchem, Bratislava, Slovakia), and 10 mmol *o*-dianisidine dihydrochloride (Sigma-Aldrich, St. Louis, MO, USA) in 25 sulfuric acid (Sigma-Aldrich, St. Louis, MO, USA). Standards (H_2_O_2_) and the samples of seminal plasma were transferred in doubles to a 96-well plate in a volume of 35 μL. Reference reading at 560 nm using Glomax Multi+ Detection System plate reader (Promega, Madison, WI, USA) was realized following the addition of 225 μL TOS R1. After 10 min incubation, 11 μL TOS R2 was added to each well, and the absorbance was spectrophotometrically assessed at the same wavelength after 3 min incubation.

### 2.11. Lipid Peroxidation (LPO)

Lipid peroxidation (LPO) was determined by the quantification of malondialdehyde (MDA) production using the TBARS assay, which was modified for a 96-well plate and ELISA reader. Samples were treated with 5% sodium dodecyl sulfate (SDS; Sigma-Aldrich, St. Louis, MO, USA) and exposed to 0.53% thiobarbituric acid (TBA; Sigma-Aldrich, St. Louis, MO, USA) dissolved in 20% acetic acid adjusted with NaOH (Centralchem) to pH 3.5, and afterward boiled at 90–100 °C for an hour. Consequently, the reaction was stopped after placing the samples on ice for 10 min. The samples were centrifuged at 1750× *g* for 10 min, and the supernatant was used for measurement of the end-product—MDA. Using the Multiskan FC microplate photometer (Thermo Fisher Scientific Inc., Waltham, MA, USA), the samples were measured at 530–540 nm [48]. The concentration of MDA is expressed as μmol/g protein.

### 2.12. Biochemical Analysis of Seminal Plasma

Biochemical quantification of parameters total proteins (TP), albumins (Alb), cholesterol (Chol), and triacylglycerides (TAG) was realized using DiaSys (Diagnostic Systems GmbH, Holzheim, Germany) commercial kits. The measurements were performed according to the manufacturer’s recommendations employing a semi-automated clinical chemistry analyzer Randox RX Monza (Randox Laboratories, Crumlin, UK).

### 2.13. Statistical Analysis

All the statistical analyses were carried out using GraphPad 8 software (GraphPad Software Inc., San Diego, CA, USA). Descriptive analysis included calculation of mean, standard deviation (SD), minimum (Min), and maximum (Max) values. All obtained data were tested for normal Gaussian distribution using a D’Agostino–Pearson normality test and Shapiro–Wilk normality test. Pearson correlation between markers of OS and chemical elements was performed to propose the possible mechanism of action of chemical elements. Furthermore, the relationship between spermatozoa quality parameters and components of seminal plasma was also evaluated using Pearson correlation. Significance was determined at *p* < 0.05 (a) and at *p* < 0.01 (A). Heatmaps with clustering were performed to visualize interactions (Pearson correlations coefficients—*r*) of chemical elements and markers of OS on spermatozoa quality.

## 3. Results

### 3.1. Sperm Quality

The stallion semen is characteristic of a high volume and lower spermatozoa concentration. The concentration in the subjected samples was 0.24 × 109/mL. The detected motility in fresh ejaculates was not of adequate quality (58.61%) and even dropped down to 47.22% after an hour of incubation. Interestingly, progressive motility in the initial measurement showed 22.20%, but in PRO_1, it was raised by almost ten percentual points to 32.57%. Spermatozoa velocity in VCL_0 was recorded at 186.24 μm/s and fell down to 121.67 μm/s throughout the incubation period. Parameters BCF and ALH exhibited remarkable variations throughout the cocultivation with SP. While the BCF decreased from 32.37 to 3.37 Hz, ALH raised from an initial 5.19 μm to an eventual 27.41 μm. A test visualizing intact and damaged spermatozoa was employed to evaluate the percentage of DNA fragmented sperm cells (10.99%). Regarding MTT assay, an average value of 0.28 Abs was calculated out of the measured range of values 0.14–0.63 Abs. Determination of live, apoptotic, and dead spermatozoa was performed by flow cytometry, using fluorescent probes AnV-FITC and PI. Mean values indicate the percentage of live spermatozoa at nearly 78%. Apoptotic spermatozoa were present in examined ejaculates in 2.77%, while dead spermatozoa were detected in 19.55% of all cells (Table 1).

### 3.2. Markers of Oxidative Stress in Seminal Plasma

Parameters evaluating the antioxidant and prooxidant status of the SP (displayed in Table 2) showed a TOS value at a level of 0.04 μmol H_2_O_2_/g TP while an extremely wide range of FRAP resulted in a mean value of 25.19 μmol Fe^2+^/g TP. A higher variation of values was detected in GPx activity; however, a higher enzyme activity was monitored for SOD. The degradation of lipids was examined by quantification of the MDA production detected in the quantity of 1.89 µmol/g TP.

### 3.3. Biochemical Composition of Stallion Seminal Plasma

Table 3 describes the protein and lipid profile of the stallion SP. The protein profile of the stallion SP included 18.59 g/L proteins out of which 2.25 g/L were Alb. Lipids in the form of Chol were present in 0.59 mmol/L, while the content of TAG was 0.82 mmol/L.

### 3.4. Chemical Composition of Stallion Seminal Plasma

Based on the concentrations in Table 4, elements fit in to the following order: Na ˃ K ˃ Ca ˃ Zn ˃ Mg ˃ Fe ˃ Cu ˃ Pb ˃ Hg ˃ Cd. The highest content out of all measured chemical elements was represented by Na (3.47 g/kg). Elements of the lowest concentrations were Cd (15.79 μg/kg) and Hg (18.87 μg/kg), recording similar values.

### 3.5. Correlation Analyses

**Figure 2:**
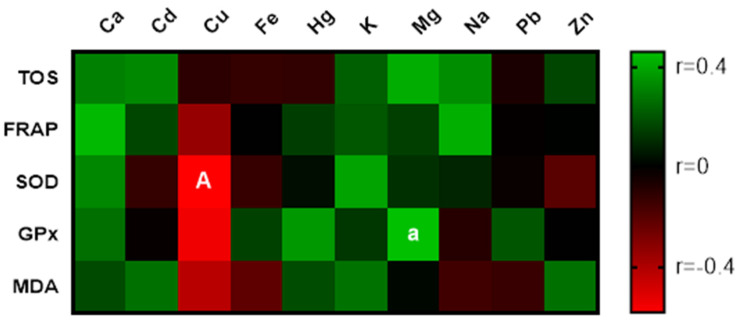
Correlations: spermatozoa parameters vs. markers of OS, biochemical parameters, and chemical elements. a—significant at *p* < 0.05; A—significant at *p* < 0.01.

Correlation analysis of chemical elements and markers of OS revealed a significantly (*p* < 0.01) negative correlation between Cu concentration and SOD activity. Relationships with FRAP, GPx, and MDA were also negative; however, the statistical significance was not observed. Other metals implicated low or medium correlations with OS parameters. The effect of biogenic elements was positive. The statistically significant correlation (*p* < 0.05) was recorded between Mg and GPx activity displayed in Figure 2.

**Figure 3:**
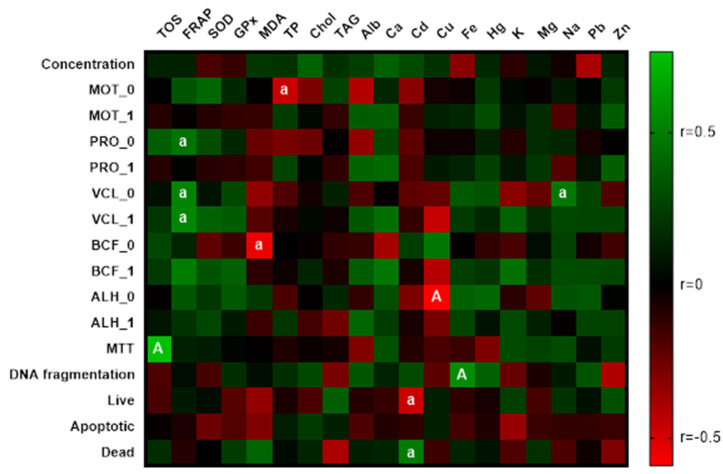
Correlations: markers of OS vs. chemical elements. a—significant at *p* < 0.05; A—significant at *p* < 0.01.

Relationships between OS markers and the spermatozoa quality (Figure 3) indicate a statistically proven benefit (*p* < 0.05) of the antioxidant activity on PRO and VCL of stallion spermatozoa. The positive correlation between TOS and MTT contended a significance at level *p* < 0.01. The level of malondialdehyde signified an important (*p* < 0.05) negative correlation with BCF_0. The same statistical significance was observed between TP and MOT_0. Concerning metals, Cd negatively (*p* < 0.01) affected the viability of cells. A significant (*p* < 0.01) interaction between Cu and ALH_0 and Fe and the percentage of spermatozoa with fragmented DNA demonstrated the toxic effect of these two metals. Biogenic elements showed a slightly positive effect on the sperm quality. The only exception was sodium. Its correlation with the positive direction was statistically significant at level *p* < 0.05.

## 4. Discussion

The role of SP in equine reproduction is a controversial topic. The fact that SP is being removed before cryoconservation implies a better longevity of spermatozoa without the SP. Therefore, the present study aimed to explore the effect of SP and its components on the sperm quality during chilled storage. In the majority of cases, stallion seminal plasma produces or induces the production of toxic hydrogen peroxide, resulting in chromatin damage, as demonstrated by Morell et al. [49]. Alghamdi et al. [20] oppositely argue that SP may serve as an important enhancer of fertilization due to the inflammatory action of SP proteins. Halo et al. [6] claim the need for the use of the commercial medium to maintain or even elevate spermatozoa motility traits in cooled horse semen. As demonstrated in the present study, the preservation of motility during the first sixty minutes after the collection was not satisfactory, requiring further investigation of the reason for a rapid motility decrease.

Copper detected in a concentration of 0.97 mg/kg negatively correlated with markers of OS. The statistical significance (*p* < 0.01) of the correlation between Cu and SOD was stated. As highlighted by Sotler et al. [50], Cu in free form may demonstrate pro-oxidant activity. These findings correspond with those proclaimed by Halo et al. [6] where a significant correlation of Cu (1.20 mg/kg) and ROS generation was emphasized. Gamal et al. [51] determined associations between Cu and spermatozoa quality and credited them to the ROS activity. Massanyi et al. [52] detected Cu in stallion ejaculate in a concentration of 0.86 mg/kg and determined the possible effect of Cu on morphological damage to the flagellum. Copper also negatively correlated with ALH_0 at *p* < 0.01, which is identical with the results of Usuga et al. [53]. Their study also reports low total motility reaching on average 65.91%. As mentioned by Kareskoski and Katila [4], semen with a higher content of Cu and Fe exhibits a lower spermatozoa motility; also, in the present study, Fe displayed a positive correlation (*p* < 0.05) with DNA fragmentation. Iron in the form of Fe^2+^ can induce lipid peroxidation, which alters spermatozoa membranes and produces MDA [54]. DNA damage is strongly related to OS [55,56]. Despite the low concentration of Cd (15.79 μg/kg), the present study suggests its deleterious effect on the spermatozoa. Significant correlations (*p* < 0.05) of Cd with the presence of live (negative correlation) and dead spermatozoa (positive correlation) were noticed. A comparatively higher concentration of Cd (90 μg/kg) was previously reported and associated with flagellum separation [52]. The effect of other metals was either mild or lacked statistical significance. Current results offer the chain reaction where the mode of action lies in the synergism of toxic metals, which induces ROS generation and subsequent degradation processes, resulting in the sperm death.

Concerning the biogenic elements, only Ca and Na show a clear direction of correlation. Both elements correlated positively with spermatozoa motility traits; however, only interaction between Na and VCL_0 was significant (*p* < 0.01). Calcium in horse semen has a positive outcome on both the concentration and motility of spermatozoa [6,8], although Pesch et al. [57] mention the importance of distinguishing between total and ionized calcium and underline the fact that 60–70% of stallion calcium is ionized. The importance of the Ca^2+^ in proper sperm physiology is projected in the several Ca^2+^-dependent enzymes and Ca^2+^ channels. The most studied sperm-specific channel CatSper (cation channel of sperm) mediates Ca^2+^ influx into sperm. Then, the sperm movement is modulated in response to the changes of intracellular calcium levels [58,59].

Mitochondrial activity was according to the correlation analysis strongly (*p* < 0.01) linked with TOS. The presence of ROS in semen has both beneficial and adverse effects. Levels of ROS within the physiological range initiate an acrosome reaction and tyrosine phosphorylation [60]. On the other hand, the excessive presence of ROS in the ejaculate causes a decrease in mobility and commences the LPO [61]. The measured activity of enzymes SOD and GPx was 1.28 U/mg TP and 22.53 U/g TP, respectively. Ball [54] highlights the importance of the GPx due to H2O2 being a bigger threat for stallion spermatozoa than superoxide anions, which are catabolized by SOD. According to the statistical analysis, the MDA level contributed to the disruption of BCF_0.

The effect of lipids present in the stallion SP was not statistically demonstrated; nevertheless, the TP content negatively correlated with the spermatozoa motility. Proteins present in stallion SP participate in essential steps of the fertilization [62]. The tremendous role of proteins in horse SP is fully manifested in the female reproductive tract. SP proteins may interact with the uterine epithelium and initiate the reaction of the female immune system. Ultimately, this primary inflammatory response cleans up the intrauterine lumen from foreign cells and possible pathogens to prepare the uterus for the descending embryo [4,18].

The semen is non-invasively accessible body fluid that may serve a breeder as an indicator of the environmental pollution, oxidative stress, DNA damage, etc. However, more extensive studies need to be carried out for the establishment of the reference values for the parameters monitored in the present study.

## 5. Conclusions

The controversy about the effect of stallion SP has been discussed over decades. Based on the accessible literature, the mode of action of SP differs in natural mating and artificial insemination. The concentration of chemical elements in the present study displayed in descending order Na ˃ K ˃ Ca ˃ Zn ˃ Mg ˃ Fe ˃ Cu ˃ Pb ˃ Hg ˃ Cd. Employing correlation analyses, regarding markers of OS and biochemical components in SP implies a notable conclusion. The findings of the present study offer the basic scheme where Cu, Fe, and Cd collectively affect redox status, thus causing DNA fragmentation and eventually cell death. While Cu negatively correlated with markers of OS, Fe and Cd were directly involved in the impairment of DNA and cell apoptosis, respectively.

## Figures and Tables

**Table 1:** Stallion spermatozoa parameters.

	Mean	SD	Min	Max
Concentration (10^9^/mL)	0.24	0.08	0.11	0.37
MOT_0 (%)	58.61	12.41	28.45	77.50
MOT_1 (%)	47.22	15.32	29.56	78.43
PRO_0 (%)	22.20	8.73	10.36	43.55
PRO_1 (%)	32.57	18.14	10.89	68.42
VCL_0 (µm/s)	186.24	25.89	140.90	218.84
VCL_1 (µm/s)	121.63	36.88	78.82	179.07
BCF_0 (Hz)	32.37	2.11	27.42	35.23
BCF_1 (Hz)	3.37	0.85	2.39	4.72
ALH_0 (µm)	5.19	0.59	4.18	6.02
ALH_1 (µm)	27.41	2.67	23.50	31.96
MTT (Abs)	0.28	0.11	0.14	0.63
DNA fragmentation (%)	10.99	4.14	3.62	20.00
Live (%)	77.68	7.64	63.08	90.85
Apoptotic (%)	2.77	1.35	0.69	5.85
Dead (%)	19.55	7.56	8.46	34.76

MOT_0—motility in time 0; MOT_1—motility after 1 h storage at 6 °C; PRO_0—progressive motility in time 0; PRO_1—progressive motility after 1 h storage at 6 °C; VCL_0—velocity curved line in time 0; VLC_1—velocity curved line after 1 h storage at 6 °C; BCF_0—beat cross frequency in time 0; BCF_1—beat cross frequency after 1 h storage at 6 °C; ALH_0—amplitude of lateral head displacement in time 0; ALH_1—amplitude of lateral head displacement after 1 h storage at 6 °C; MTT—mitochondrial toxicity test.

**Table 2:** Markers of oxidative stress in stallion seminal plasma.

	Mean	SD	Min	Max
TOS (μmol H_2_O_2_/g TP)	0.04	0.02	0.01	0.12
FRAP (μmol Fe^2+^/g TP)	25.19	11.88	0.75	42.93
GPx (U/g TP)	22.53	6.34	15.82	42.79
SOD (U/mg TP)	1.28	0.62	0.51	3.38
MDA (µmol/g TP)	1.89	0.92	1.02	4.47

TOS—total oxidant status; FRAP—ferric-reducing ability of plasma; GPx—glutathione peroxidase; SOD—superoxide dismutase; MDA—malondialdehyde.

**Table 3:** Selected biochemical components of stallion seminal plasma.

	Mean	SD	Min	Max
TP (g/L)	18.59	5.34	8.06	30.85
Alb (g/L)	*2.25*	0.57	1.41	3.18
Chol (mmol/L)	0.59	0.05	0.53	0.71
TAG (mmol/L)	0.82	0.60	0.24	2.24

TP—total proteins; Alb—albumins; Chol—cholesterol; TAG—triglycerides.

**Table 4:** Chemical elements detected in stallion seminal plasma.

	Mean	SD	Min	Max
Ca (mg/kg)	169.90	62.59	72.00	288.00
Cd (μg/kg)	15.79	25.23	10.00	120.00
Cu (mg/kg)	0.97	0.34	0.26	1.72
Fe (mg/kg)	2.60	2.46	1.32	11.42
Hg (µg/kg)	*18.87*	*2.81*	14.29	25.52
K (g/kg)	0.91	0.12	0.62	1.12
Mg (mg/kg)	43.85	13.89	24.07	69.04
Na (g/kg)	3.47	0.34	2.84	4.08
Pb (mg/kg)	0.12	0.04	0.107	0.280
Zn (mg/kg)	64.51	7.84	48.40	77.00

## Data Availability

Not applicable.
